# Modern Methods for Preventing the Progression of Myopia in Children

**DOI:** 10.3390/jcm15124734

**Published:** 2026-06-18

**Authors:** Zofia Pniakowska, Sonia Czarkowska, Natasza Kurys, Maria Orłowska, Piotr Jurowski

**Affiliations:** 1 Department of Ophthalmology and Vision Rehabilitation, Medical University of Lodz, 90-549 Lodz, Poland; sonia.czarkowska@stud.umed.lodz.pl (S.C.); natasza.dzieza@gmail.com (N.K.); piotr.jurowski@umed.lodz.pl (P.J.); 2 Optegra Eye Clinic, 90-127 Lodz, Poland

**Keywords:** myopia, myopia in children, progression of myopia, specialized lenses, orthokeratology

## Abstract

The progression of myopia in the pediatric population is currently highly prevalent. Thus, there is a need to look for new, effective methods that might suppress this pathological process. It not only affects visual comfort but also increases the risk of developing further ocular complications. The aim of the study is to review the literature and summarize contemporary methods for preventing the progression of myopia in children. The review is based on publications available on PubMed from the past 17 years, supplemented by the current literature on advanced digital technologies in ophthalmology. This article highlights that among other treatments such as orthokeratology, low-dose atropine or specialized lenses, there are also further beneficial options, including increased outdoor time, reduced screen time or the implementation of the latest medical innovations. The results indicate that defocus spectacle lenses may reduce myopia progression by approximately 50–67%, while orthokeratology has been associated with about a 46% reduction in axial elongation. Although there is a broad spectrum of therapeutic strategies, it is essential to develop novel approaches to myopia prevention in children to improve their quality of life from childhood into adulthood.

## 1. Introduction

In recent decades, myopia has gone beyond being considered a common visual defect and has become a major global public health problem and a leading cause of visual impairment and blindness [1,2]. In fact, it has been identified as one of the five immediate priorities for the World Health Organization’s (WHO) “Vision 2020” initiative aimed at eliminating preventable causes of blindness [3]. This “myopia boom” is a growing international concern [1]. Current trends suggest that by 2050, there will be nearly 4.76 billion people worldwide with myopia, including 938 million with high myopia [1]. The increase is particularly dramatic in East Asia, where the condition already affects 80–90% of eighteen-year-olds, with approximately 10–20% of them diagnosed with high myopia [1].

The primary pathological mechanism of myopia is the excessive elongation of the eye, which causes light to focus in front of the retina rather than directly on it [2]. High myopia, typically defined as a refractive error exceeding −5.00 diopter (D) or −6.00 D under relaxed accommodation, is associated with a significant risk of irreversible blindness [1,2,4]. This permanent vision loss is often caused by serious complications, including glaucoma, retinal detachment, and myopic macular degeneration [1,2,4].

Myopia is a refractive error with a complex etiology involving both genetic predisposition and environmental factors [2]. Genetics plays a crucial role—children with two myopic parents face a 60% risk of developing the condition, whereas those without a familial history have only a 20% risk [2]. The genetics of myopia is highly complex, with inheritance being polygenic rather than driven by a single factor [2].

The penetrance of individual single-nucleotide polymorphisms (SNPs) remains low [1]. Overall, all known genetic variants combined only explain about 18.4% of the heritability variance [1].

This suggests that while genetics creates a predisposition, the current myopia epidemic is primarily triggered when these internal factors meet adverse environmental conditions [1,2]. Research points to two primary mechanisms driving this interaction. Firstly, environmental factors such as intensive education and prolonged near work can induce epigenetic changes that modify gene expression, effectively activating dormant genetic predispositions [2]. Secondly, spending time outdoors serves an important protective function, as exposure to natural light stimulates the release of dopamine in the retina [2,4]. This neurotransmitter prevents excessive elongation of the eyeball’s axis, while limiting exposure to sunlight during the day disrupts metabolic pathways and weakens the mechanism that inhibits the development of myopia [2,4]. This allows the genetic predispositions to myopia to fully manifest [1,2]. Spending time outdoors has a strong preventive effect—each additional hour of daily outdoor activity correlates with an approximately 20% reduction in myopia incidence [2]. However, comprehensive systematic reviews highlight a crucial clinical distinction: while additional outdoor time effectively lowers the incidence of myopia onset, it does not appear to slow the progression of the condition once it has already developed [3].

Among these environmental drivers, a lifestyle dominated by prolonged near work, such as reading, studying, and the intensive use of digital screens, significantly contributes to the rising incidence [2]. Physiologically, sustained near work often induces greater accommodative lag in myopic patients compared to nonmyopic individuals [3]. This lag results in light focusing slightly behind the retina, which acts as a powerful stimulus to increase pathological eye growth [3]. It is estimated that near-work activities increase the risk of myopia by 31% in children and 21% in adults [2]. This impact was notably intensified during the COVID-19 pandemic, leading to a phenomenon widely recognized as “quarantine myopia” [2]. During this period, the sudden shift to digital learning, combined with extended home confinement and a severe reduction in outdoor engagement, drastically accelerated the progression of the condition, particularly in young school-aged children [2].

The clinical importance of preventing excessive ocular growth is to reduce the risk of permanent vision loss associated with pathological myopia. Because lifestyle modifications alone may be insufficient to stop progression in already myopic children, given the increasing number of patients at risk, this article aims to review the most effective treatment strategies [3]. In the following sections, lifestyle interventions, modern optical treatments such as defocus incorporated multiple segments (DIMSs) and highly aspherical lenslet target (HALT) lenses, and pharmacological options such as low-dose atropine as well as emerging innovations such as low-level red light (LRLR) therapy and AI-driven digital twins, are assessed to provide a current perspective on the management of myopia in children.

During the preparation of this manuscript, the authors utilized artificial intelligence tools solely for English-language editing, stylistic refinement, and proofreading to ensure academic standards. All scientific content, literature selection, clinical interpretations and final conclusions were prepared, verified and approved by the authors.

## 2. Treatment

### 2.1. Lifestyle Changes

#### 2.1.1. Outdoor Activities

Many studies show that spending more time outdoors is associated with a lower risk of myopia in school-age children. Randomized trials from China and Taiwan demonstrate that adding time spent outdoors during the school day slightly slows progression of myopia and significantly reduces new cases, especially among children who do not have myopia yet [5]. Ensuring children spend at least 80 min outdoors each day has been linked to a reduction in myopia estimated at almost half, while classroom measures alone (such as better lighting or scheduled breaks) have little impact [6].

Evidence suggests that the protective benefits depend primarily on the total time spent outdoors rather than on sports, as indoor physical activity does not appear to reduce the risk of myopia [7]. Studies show that 2–3 h outdoors daily is the most effective [8]. A large prospective study further showed that increased outdoor exposure reduces the progression of myopic and axial elongation, with better effects observed at a higher light intensity and longer exposure time [9]. Ways that encourage spending time outdoors, such as sending message reminders to parents, have long-lasting benefits [10].

There are many mechanisms that may explain this effect, including exposure to bright light, slower axial growth of the eye and reduced close-up work [11]. Clinical studies showed that bright outdoor light can stimulate the retina to release dopamine. Dopamine acts as a neuromodulator, participating in the regulation of ocular growth and emmetropization. Increased dopamine activity in the retina is thought to inhibit excessive axial elongation, reducing the risk of myopia onset and progression, although the precise mechanisms remain incompletely understood [12]. While outdoor activity provides overall physical and mental health benefits, it is important to consider potential risks, such as exposure to UV radiation. Currently, it is considered that visible light is beneficial for myopia prevention, not UV radiation, which shows the need for sustainable and safe outdoor activities [6,7].

#### 2.1.2. Near Work and Screen Exposure

Behavioral methods are becoming increasingly important in preventing myopia, including, for instance, limiting close-up work and time spent in front of the screen [13]. Many studies show that prolonged looking at short distances for long periods of time, whether while reading, working at the computer or using a smartphone, increases the risk of the development and progression of this defect in children [14]. A cohort study, Generation R Study [15], observed that there is a link between increased computer use and myopia in 9-year-olds. Similar results were confirmed by studies conducted in China [16], in which longer screen time was associated with a higher incidence of myopia; however, the strength of association varies across studies.

The mechanism of this phenomenon is explained by excessive accommodative tension and elongation of the eyeball axis when working at close range [17], as well as by a smaller font and shorter viewing distance in the case of mobile devices [18]. Limited exposure to daylight is also important, as it is a protective factor in the emmetropization, probably by influencing dopaminergic transmission in the retina [19].

During the COVID-19 pandemic, when learning moved to homes, there was an increase in the incidence and progression of myopia, especially in young children [20]. Review studies show that most studies support an association between screen time and higher risk of myopia [21]. Simultaneously, it has been shown that school programs that limit screen time and promote breaks can effectively slow the progression of this defect [22]. Despite consistent epidemiological links, the relationship remains complex and disturbed by lifestyle and educational factors. Therefore, limiting screen time should be considered as part of a broader behavioral approach, rather than as a standalone intervention.

### 2.2. Optical Methods

Most contemporary optical interventions for myopia are based on the concept of peripheral retinal defocus. Hyperopic peripheral defocus occurs when light rays focus in the peripheral retina behind the retinal plane, generating a signal that can stimulate axial elongation of the eye. Myopic peripheral defocus, on the other hand, occurs when peripheral light rays focus in front of the retina. This optical signal inhibits excessive eye growth and slows the progression of myopia.

Lenses used to control myopia progression can be divided according to their mechanism of action into those generating peripheral myopic defocus (spectacles and contacts) and those modifying the shape of the cornea (orthokeratology).

#### 2.2.1. Defocus Generating Spectacle Lenses

Current optical approaches to myopia control are based on the hypothesis that peripheral retinal image quality influences ocular growth. According to this model, peripheral hyperopic defocus may stimulate axial elongation, whereas peripheral myopic defocus may act as a growth-inhibiting signal. However, the role of peripheral defocus remains controversial, as some studies have failed to demonstrate that relative peripheral hyperopia predicts the subsequent onset or progression of myopia. Therefore, although peripheral defocus remains a useful therapeutic concept, its precise role in the pathogenesis of myopia has not been fully established. In classic single-focal lenses, there is good central focus; however, in peripheral parts of the retina, relative hyperopic defocus may occur, which can promote axial elongation of the eye. The correlation between the peripheral refraction profile and the rate of myopia progression has been observed in studies analyzing the influence of relative peripheral hyperopia on the development of the defect [23]. In a large prospective cohort study by David A. Atchison et al. [24], it was assessed if the presence of relative peripheral hyperopia (RPH) could be a predictive factor of the development and progression of myopia in children. The analysis included 1714 children around 7 years old and 1063 adolescents around 14 years old. Central and peripheral refraction were measured under cycloplegia at eccentricities up to ±30°, and the follow-up lasted two years (younger cohort) and one year (older cohort). The study results showed no significant relationship between relative peripheral hyperopia and the subsequent development of myopia or the rate of its progression. Children who developed myopia during follow-up did not have a higher RPH at baseline than children who remained emmetropic. Moreover, it was not confirmed that the size of the peripheral hyperopic defocus was a predictive factor of changes in the spherical equivalent over time. Furthermore, the observed peripheral hyperopia may be a consequence of geometric changes in the eyeball in the course of myopia rather than the primary factor responsible for its development [24].

Modern contact lenses introduce controlled myopic defocus in the peripheral area by applying many microlenses or segments with positive optical power placed around the central correction zone. This design enables two optical signals to reach the retina, one that is a sharp central image and a second one, which is intentionally created defocus in the periphery. The study performed by Han Yu Zang et al. [25] analyzed the effect of using DIMS lenses for two years on the peripheral refraction profile and the rate of myopia progression in children. The lens design included a central monofocal zone of about 9 mm in diameter, surrounded by a ring with many hundred microlenses with a positive power of about +3.50 D. These segments were placed regularly in the peripheral area, which caused focusing part of the light rays in front of the retina in its peripheral regions. The study involved 183 children aged 8–13 years who were randomly assigned to wear DIMS lenses or classic single-focal lenses. Central and peripheral refraction were rated under cycloplegia at horizontal eccentricities up to ±30°, and measurements were performed every six months for two years. In the study, it was observed that in children using DIMS lenses, the peripheral refraction profile shifted toward a more myopic direction compared to a control group, whereas in the group wearing monofocal lenses, an increase in relative peripheral hyperopia was observed with progression of the defect. At the same time, less myopia progression was confirmed in the DIMS group—both in terms of change in the spherical equivalent and axial elongation of the eyeball. During the two-year follow-up period, the reduction in progression was noticeable compared to monofocal correction [25].

A meta-analysis including studies on various constructions of DIMS showed that the average reduction in the progression of myopia in comparison to single-focal correction was about 50% while also reducing the axial elongation of the eye. However, the effect depends on regular and long-hour wearing of lenses [26].

Apart from DIMS, other spectacle lens designs generating peripheral myopic defocus have been produced, such as HALT used in Essilor Stellest and Defocus Incorporated Soft Segments technology in Hoya MiyoSmart. HALT lenses are made from 1021 adjacent aspherical lenses arranged in concentric rings around a central clear zone, creating a volumetric myopic defocus rather than point defocus. In a randomized controlled trial performed by Bao et al., it was observed that children wearing Stellest lenses had around a 67% reduction in myopia progression (spherical equivalent) and about a 60% reduction in axial elongation vs. children using single-vision lenses [27]. Although HALT lenses have demonstrated impressive efficacy, comparisons between studies should be interpreted with caution due to differences in study design, outcome measures, and baseline characteristics of the children studied. Direct comparative studies comparing HALT technology with other modern myopia-correcting lenses remain limited. Similarly, CARE (Cylindrical Annular Refractive Elements) lenses use multiple annular segments of positive additive power to produce peripheral myopic defocus while maintaining central visual quality. In clinical studies, it was demonstrated that this design was also slowing the axial elongation and progression of myopia compared to single-vision correction [28]. It is important to emphasize that in most studies, no deterioration in distance vision acuity or some adaptation problems were observed, and the reported visual impairment had a temporary effect [25]. This means that these lenses can be used in the future as a first-line method in children with progressive myopia, especially in the younger age, when the rate of axial elongation is the highest.

Despite encouraging results, there are several limitations. Most DIMS studies have been conducted in East Asian populations, where the frequency and progression of myopia differ from those observed in European populations. Furthermore, follow-up periods exceed three to six years, limiting conclusions regarding the long-term efficacy and durability of treatment effects. Furthermore, treatment success is highly dependent on 24 h spectacle wear, which may be more difficult to achieve in real-world settings than in controlled clinical trials.

#### 2.2.2. Soft Multifocal Contact Lenses

Soft multifocal contact lenses are intended to control myopia and use a similar rule as defocus spectacle lenses. However, due to direct adhesion to the cornea, they enable stable mapping of optical zones relative to the axis of vision. Dual-focus designs have a central zone providing full-distance correction and concentric positive power rings that generate myopic defocus in the peripheral area of the retina [29].

In a 3-year randomized controlled trial, the influence of soft contact lenses with dual focus used every day on the progression of myopia in children was observed. In the study were included children aged 8–12 with myopia ranging from −0.75 to −4.00 D and astigmatism below 1.00 D, and who had no previous experience with contact lenses. Participants were randomly selected into two groups, one with soft contact lenses MiSight 1 day with a dual-focus design and another with standard, single-focal contact lenses made from the same material. The first results regarding the change in spherical equivalent (SE) and axial extension (AL) were measured once a year for 3 years during cycloplegia. It was demonstrated that children wearing dual-focus lenses experienced slower progression of myopia than children in the control group. The average change in SE in patients from the experimental group was lower by about 0.73 D compared to the control group (which is about a 59% reduction in myopia progression) and axial elongation was reduced by 0.3 mm, which corresponded to a reduction in axial growth of more than 50% compared to the control group. Furthermore, the results showed that the effectiveness of this therapy was maintained throughout the study, which suggests that regular use of this form of optical intervention provides a lasting therapeutic effect [30]. A limitation of contact lens research is the potential impact of adherence. Clinical trial participants often receive extensive supervision and support, which may result in better adherence than typically observed in routine clinical practice. Consequently, real-world effectiveness may be lower than that reported in randomized controlled trials.

EDOF (extended depth of focus) designs are based on more complex modeling of higher-order aberrations and extending the depth of the focus range. This leads to the modulation of the optical signal reaching the retina in a different way than classical division into correction and defocus zones. This strategy in limiting the progression of myopia was confirmed in comparative studies, which showed a slowing in the progression of the defect compared to standard single-focus lenses [31].

In the study by Alagsam et al., various optical designs, such as dual-focus lenses, progressive addition (center-distance multifocal) designs, and EDOF designs, were compared with classic single-focal lenses or single-focal glasses as a control group. It was observed that the mean reduction in the progression of myopia measured by the change in SE was in the range of about 30–60% depending on the optical design, observation time and addition parameters. The average rate of progression in the test group was lower than in the control group. Similar effects were observed in the axial elongation of the eye, as the reduction in the increase in the axial length was on average about 0.1–0.3 mm over the 1–3 years. Moreover, the most predictable and stable effects were provided by dual-focus lenses, while in progressive addition designs, the effect was more varied, which can result from differences in the size of the addition zones, addition values and individual lens centration on the eye. EDOF designs had a medium, but significant reduction in progression. Furthermore, the effectiveness of the therapy was greater in younger children, especially in the first stage of the development of myopia. Early intervention was associated with a bigger reduction in the rate of progression compared to children, who started treatment at a later age. The other factor influencing the effectiveness of treatment was patients’ compliance about the time of wearing lenses. In studies reporting daily use for >6–8 h, the therapeutic effect was greater than in analyses with patients with irregular use. The authors highlighted the average heterogeneity of the studies resulting from differences in methodology, length of follow-up, age of patients and lens design, but apart from those limitations, the overall effect was that soft multifocal lenses slow down the progression of myopia compared to single-focal correction [32].

In practice, contact lenses provide a wider field of view and do not cause prismatic effects typical for glasses; however, they need greater care from patients, especially in terms of hygiene.

### 2.3. Orthokeratology

Orthokeratology (OK) is an established method of myopia control that involves wearing specially designed, rigid gas-permeable (RGP) contact lenses with reverse geometry overnight [2,4]. The primary mechanism is the mechanical remodeling of the corneal shape during sleep, which flattens the central cornea and steepens the mid-peripheral area [2,4]. This change in corneal curvature eliminates peripheral hyperopic defocus and creates peripheral myopic defocus, which serves as a powerful stimulus to inhibit excessive increase in axial length (AL) [2,4]. By using lenses at night, patients achieve clear vision during the day without traditional spectacles, which is beneficial, especially for physically active children [2]. As noted in Cochrane systematic reviews, because these lenses temporarily reduce the myopic refractive error during the day, their true disease-modifying effect on myopia progression can only be accurately measured by assessing AL elongation rather than refractive changes [3].

Numerous clinical trials demonstrate that OK effectively reduces AL progression in pediatric populations, though these data must be interpreted with caution due to a high risk of bias, mainly due to a significant percentage of participants withdrawing from the study [2,3,4]. For instance, a two-year Longitudinal Orthokeratology Research in Children (LORIC) clinical study in Hong Kong reported an average AL increase of only 0.29 mm in the OK group compared to 0.54 mm in the control group—a 46% reduction [2]. Comparable studies conducted in the United States and Japan reported limited AL growth of 0.32 mm and 0.39 mm, respectively, in children using OK compared to those in control groups [2]. In a Spanish study of children aged 6 to 12, the OK group experienced a 0.47 mm increase in AL versus 0.69 mm in the control group, which reflects a 36% slowing of myopia progression [2].

The benefits of OK are also applicable to children with corneal astigmatism [2]. Toric OK lenses are recommended for patients with astigmatism exceeding 1.50 D to ensure better lens centration and clear daytime vision without the need for glasses [2]. The Toric Orthokeratology for Slowing Eyeball Elongation (TO-SEE) study in Hong Kong reported a mean AL growth of 0.30 mm over two years in astigmatic children treated with OK lenses, compared to 0.64 mm in the control group, representing a 52% reduction in axial elongation for this population [2,4]. Although these individual findings from East Asia are very encouraging, their practical applicability to broader global populations may be limited [1,4]. It may be due to the fact that most efficacy data come from a specific demographic region with an exceptionally high prevalence of myopia. Furthermore, the reliance on relatively short follow-up periods (usually two years) in landmark randomized controlled trials limits our understanding of the long-term durability of this therapeutic effect [3]. These local data are somewhat tempered by the broader Cochrane meta-analyses. Although they support moderate-certainty evidence for efficacy, they show a more conservative cumulative pooled mean AL reduction of 0.28 mm over two years compared to single-vision lenses [3]. This highlights the variability across study designs and centers.

Recent meta-analyses, backed by the International Myopia Institute (IMI) reports, show that a combination therapy of OK paired with 0.01% atropine drops delivers better results than using either treatment alone (Table 1) [2]. Data indicate that this dual approach provides an additional 0.12 mm reduction in AL elongation compared to OK monotherapy [2]. However, the long-term safety, optimal dosing regimens and synergistic mechanisms of this dual approach remain subject to ongoing international clinical trials, as current evidence is still limited by short follow-up periods [4]. Moreover, the high clinical effectiveness of both monotherapy and combined OK therapy is largely dependent on adherence to treatment and strict hygiene, which creates compliance challenges in pediatric care [2]. The clinical reality is that overnight contact lens wear carries risks ranging from mild conjunctival hyperemia and corneal staining to more severe complications like papillary conjunctivitis [2]. Although the risk of serious microbial keratitis in pediatric patients wearing OK lenses is relatively low—estimated at 13.9 per 10,000 patient-years (comparable to the safety profile of extended-wear soft contact lenses)—and Cochrane reviews indicate that young children are less likely to suffer severe corneal ulcers than college-aged adults, the potential for sight-threatening complications requires strict patient selection [3,4]. It is also crucial to recognize the consequences of ceasing the combined treatment [2,4]. Because the corneal structural changes induced by OK are reversible, discontinuing contact lens wear returns the eye to its natural rate of myopia progression [2,4]. However, concomitant discontinuation of atropine use can cause a rebound effect, inducing an unnaturally accelerated progression that could potentially negate previous therapeutic benefits [2,4].

### 2.4. Clinical Implications and Practical Recommendations

Lifestyle changes, such as increasing outdoor exposure to at least two hours per day and limiting activities related to work, should be recommended for all children, regardless of their refraction, and be as important as the early identification of children at risk of progressive myopia and requiring suitable treatment.

Optical interventions should be considered in children with documented progressive myopia. Defocus spectacle lenses may be preferred as a first-line therapy in younger children due to their better safety profile and ease of use. Soft multifocal contact lenses offer an alternative for more motivated patients who can maintain adequate lens hygiene. Orthokeratology may be an option for physically active children or those who wish to avoid wearing glasses during the day.

Regular follow-up examinations should include cycloplegic refraction and axial length measurements, if available. The choice of treatment should be individualized depending on the patient’s age, rate of progression, lifestyle, ocular characteristics, compliance with medical recommendations, and family preferences.

### 2.5. Evidence Hierarchy and Clinical Framework

According to international consensus, including those of the IMI, myopia treatment should be stepwise and evidence-based. Behavioral interventions, such as increased outdoor exposure, are widely recommended due to their preventative benefits and safety profile. For progressive myopia, pharmacological and optical interventions are the basis of treatment. Data from systematic reviews, including Cochrane analyses, suggest the highest level of certainty for optical interventions based on atropine and defocus, while other approaches demonstrate moderate or increasing levels of evidence.

However, interpretation of the evidence base is limited by heterogeneity in study design, ethnicity, outcome measures, and duration of follow-up. These factors complicate direct comparisons of interventions and limit the development of universal treatment algorithms.

## 3. Pharmacological Treatment

### 3.1. Low-Dose Atropine

Pharmacological interventions, particularly the topical application of low-dose atropine, are now standard in the clinical management of pediatric myopia [1,2,4]. Cochrane systematic reviews confirm that antimuscarinic medications like atropine effectively slow both myopic refractive error progression and AL growth compared to a placebo [3]. Atropine sulfate is a muscarinic receptor antagonist that interferes with acetylcholine’s role in regulating ocular growth [2]. While its exact mechanism remains under investigation, recent evidence suggests it acts through non-accommodative pathways by reducing scleral fibroblast proliferation, modulating the secretion of growth factors like transforming growth factor-beta (TGF-β), and increasing dopamine release, which collectively inhibit pathological AL increase [2].

Historically, high concentrations of atropine (0.5% and 1%) demonstrated significant efficacy [2,4]. Cochrane meta-analyses report a mean reduction in myopia progression of 1.00 D over one year compared to a placebo [3]. However, their clinical application was severely limited by adverse effects [2,3,4]. As noted in rigorous systematic reviews, children treated with these eye drops frequently experienced marked photophobia, near-vision blur due to accommodation difficulties, conjunctival papillae and follicles, and local discomfort [2,3,4]. Additionally, higher doses are associated with a pronounced “rebound effect” after treatment cessation [2,4]. This accelerated post-treatment eye growth can rapidly erase prior therapeutic gains [2].

To mitigate these limitations, clinical focus shifted toward low-dose formulations, though interpreting data from landmark trials requires strict methodological scrutiny [2,4]. The Atropine for the Treatment of Childhood Myopia 2 (ATOM2) study, which evaluated 0.5%, 0.1%, and 0.01% concentrations over a two-year treatment phase followed by a one-year washout period, revealed that while the 0.5% dose offered the greatest initial control (myopia progression of −0.30 D and AL increase of 0.27 mm over two years), the 0.01% concentration still effectively slowed progression (−0.49 D and 0.41 mm, respectively) [2,4]. More importantly, the 0.01% dose significantly minimized the rebound phenomenon—during the washout phase, the refractive error progressed by only −0.28 D (0.19 mm AL increase) compared to a drastic −0.87 D (0.26 mm AL increase) in the 0.5% group and simultaneously reduced the incidence of photophobia and accommodative loss [2,4]. Crucially, because the 0.01% group experienced minimal accelerated eye growth during the washout phase, it demonstrated the lowest overall myopia progression across the entire three-year study (totaling just −0.72 D) [2,4]. Building on these findings and addressing ATOM2’s limitation of lacking a placebo group, the randomized, double-blind Low-Concentration Atropine for Myopia Progression (LAMP) study provided crucial insights into concentration-dependent responses by comparing 0.05%, 0.025%, and 0.01% doses against a placebo control [2,4]. The results indicated that 0.05% atropine is the optimal concentration, achieving double the efficacy of the 0.01% dose over a two-year period by restricting mean myopic progression to −0.55 D (versus −1.12 D for the 0.01% group) and limiting AL elongation to just 0.39 mm (versus 0.59 mm), without a clinically significant increase in adverse effects [2,4].

A major limitation of both the ATOM2 and LAMP trials was their geographic homogeneity, as both cohorts consisted exclusively of East Asian children, populations known to exhibit faster baseline myopia progression rates than Caucasian cohorts [2,3,4]. However, this limitation has been recently addressed by international trials like the Childhood Atropine for Myopia Progression (CHAMP) study conducted in the U.S. and Europe, which successfully demonstrated that 0.01% atropine effectively slows progression across a wide, global demographic range [2]. Furthermore, long-term safety data exceeding three to five years remain sparse, and maintaining daily treatment compliance over several years poses a practical challenge for pediatric care [2,4]. Finally, while combining atropine with optical interventions like multifocal spectacles shows synergistic potential, the current evidence base remains limited by varying study designs and short follow-up periods [3,4].

### 3.2. Cyclopentolate and Pirenzepine

While low-dose atropine remains the gold standard among pharmacological interventions, other antimuscarinic agents, such as cyclopentolate and pirenzepine, have also been rigorously evaluated for myopia control [4]. Early trials investigating 1% cyclopentolate found it effective in slowing progression compared to a placebo, with one landmark study showing a progression of −0.58 D/year versus −0.91 D/year in the control group [3,4]. Similarly, pirenzepine, typically administered as a 2% ophthalmic gel, demonstrated significant efficacy in large-scale clinical trials [3]. Comprehensive Cochrane meta-analyses provide moderate-certainty evidence that pirenzepine effectively slows myopic progression, reducing refractive error change by an average of 0.31 D and limiting AL elongation by 0.13 mm over a one-year period compared to a placebo [3,4].

However, like high-dose atropine, the clinical application of these alternatives was severely hindered by ocular adverse effects [3,4]. Children treated with cyclopentolate frequently experienced near-vision blur, while pirenzepine use was predominantly associated with decreased accommodation, the formation of conjunctival papillae and follicles, and medication residue on the eyelashes [3,4]. Despite their proven clinical efficacy in modifying disease progression, the development of both pirenzepine and cyclopentolate as myopia reduction therapies was eventually abandoned [4]. Today, neither medication is considered a viable treatment option in contemporary pediatric myopia management due to their side effect profiles and the overwhelming clinical superiority of low-dose atropine [2,3,4].

## 4. Emerging and Experimental Approaches in Myopia Control

### 4.1. Low-Level Red Light Therapy

Through recent years, a new method based on LRLR has been under consideration. Such an idea has been proposed as a potential intervention that initiates a process of healing and reducing the inflammation in the body. It has been suggested that red light can penetrate into deeper tissues and stimulate cells to repair as much as commence the rebuilding process. One proposed mechanism involves the stimulation of mitochondrial activity and modulation of cellular energy metabolism [33]. Its effectiveness was questioned and tested; however, a meta-analysis from 2025 by Haobo Fan et al. suggested that LRLR therapy may delay the progression of myopia in the pediatric group of patients during the 6-to-24-month observation process. The meta-analysis obtained 20 scientific works and 2638 participants between 3 and 16 years old. The results include slower AL growth, a lower progression of spherical equivalent refraction, and a greater increase in subfoveal choroidal thickness.

The above method uses LRLR with a wavelength of 635 nm or 650 nm. A patient looks straight into the red light point inside the machine. It requires two sessions per day with a minimum of a 4 h break and repeated at least five times a week.

The meta-analysis demonstrated that, compared with the control group, the change in axial length was reduced by 0.61 mm and the progression of refractive error was lower by 1.33 D.

Some studies have suggested that the degree of the baseline myopia and the LRLR treatment frequency correlates with the effectiveness of the therapy. Although preliminary clinical evidence suggests potential benefits, there is still a risk of retinal damage, which requires further well-designed clinical trials to confirm both the efficacy and safety [34].

### 4.2. Digital Twin—Fundus2Globe

With the development of artificial intelligence, we are able to try and reach for innovative, not-used-before methods and to broaden horizons in many diverse fields including ophthalmology. There is a trial by Danli Shi et al. using Fundus2Globe that might help us successfully observe myopia progression by using 2D images of a patient’s eye and transferring them into 3D projections. That way, without using high-cost MRI imaging, it allows us to assess the details of the posterior part of the eyeball. It operates with metadata, such as spherical equivalent, AL, along with latent representations of Color Fundus Photography as the input. The trial included 167 eyeballs from 99 patients. The age range was from 12 to 67 years old and 50.5% of the participants were male. Fundus2Globe used counterfactual generation to simulate hypothetical scenarios. Topological fidelity between the generated and real eye globe was stated mainly using the chamfer distance, completed with the Hausdorff distance and point-to-surface distance to measure spatial deviations. Fundus2Globe is an experimental AI-based modeling tool designed to improve the understanding of ocular morphology and facilitate research into myopia progression. Although it might result in an improved, personal treatment plan, its clinical utility has not yet been established and requires external validation in larger prospective cohorts [35].

### 4.3. AdoRA2a

Although there is evidence that 7-methylxanthine, a caffeine metabolite, regulates adenosine receptors in myopia progression [36], there is a trial by Shengcong Liu et al. from January 2026 focusing on more targeted treatment. It concentrates on increased levels of adenosine activated by CD39 and CD79 enzymes. Monocular form deprivation myopia was induced in mice in order to measure adenosine and enzymes. Measurements showed an increasing trend. Decreasing the activity of AdoRA2a through AdoRA2a-CKO or KW6002 significantly reduced the progression of monocular form deprivation myopia (FDM) in mice without altering the physiological refractive development. The eye measurements were performed using a custom-built spectral-domain optical coherence tomography (SD-OCT) system. Importantly, as these findings refer to mice, they cannot be directly extrapolated to humans. Further studies are necessary before AdoRA2a inhibition can be implemented as a potential therapeutic strategy in pediatric myopia [37].

### 4.4. Violet Light

As the importance of spending time outside in order to prevent myopia progression was mentioned before, it is not just sun exposure that matters—it is the violet light of the 360–400 nm wavelength that plays a key role. Analysis from 2016 by Hidemasa Tori et al. highlights significant upregulation of the EGR1 gene that is responsible for myopia suppression. Due to the fact that in present times, we intensify UV protection, these findings actually suggest the potential role of violet light exposure therapy to suppress the development of refractive error. Modern prevention therefore suggests the use of special contact and spectacle lenses that selectively transmit the violet light band, stimulating the eye’s natural protective mechanisms even while indoors. However, current evidence remains limited and insufficient to support routine clinical recommendations. Additional controlled clinical studies are required to determine the efficacy and long-term safety [38].

### 4.5. Bright Light Therapy

According to what was written earlier in the article, it is beneficial to spend time outside in natural light; furthermore, there is an ongoing trial conducted by Ying Hon et al. [39] whose goal is to define the effectiveness of bright light therapy in myopia progression. Research shows that dopamine is responsible for suppressing eyeball growth; furthermore, bright light increases signaling from photoreceptors along with the intrinsically photosensitive retinal ganglion cells, resulting in elevated dopamine synthesis and release [40]. The study from 2025 was a randomized controlled trial with a double-blind design. They use bright light therapy, placebo light therapy, a DIMS spectacle lens and also a single-vision spectacle lens. The trial includes children aged 7–12 years old with refractive errors from −0.75 D to −5.00 D using natural white light with an intensity of 10,000 lux at a 30–35 cm distance between the eye and screen. Effectiveness was measured using cycloplegic autorefractometry spherical equivalent refraction and AL at six-month intervals over a two-year period. The researchers’ assumption is to enhance the strength of combining DIMS spectacle lenses and simultaneously incorporating bright light exposure. Since the trial is ongoing, conclusions regarding clinical effectiveness cannot yet be drawn. The results will help determine whether bright light therapy provides additional benefits beyond currently established myopia control strategies [40].

## 5. Discussion

The global increase in myopia is a known public health challenge, particularly among children and adolescents. Current evidence shows that while genetic predisposition contributes to the increased prevalence of myopia, environmental and lifestyle factors, such as prolonged work, excessive exposure to screens, and limited outdoor activity, play a huge role in the rise in myopia incidence. Lifestyle interventions, particularly increasing time spent outdoors, remain an essential prevention for this condition. Exposure to natural light is believed to stimulate dopamine release in the retina, which may contribute to the regulation of eye growth and can reduce the risk of myopia. However, the underlying biological pathways remain incompletely understood, and current evidence should be interpreted as associative rather than definitely causal. Among currently available treatments, optical interventions such as DIMS and HALT spectacle lenses, multifocal contact lenses, and orthokeratology were shown to be highly effective in slowing axial elongation and refractive progression in children. However, direct head-to-head comparisons are limited, and differences in study design, population characteristics, and follow-up duration complicate the interpretation of cross-over study results. Efficacy appears to be greatest when treatment is initiated early and adherence is high; however, in practice, adherence is often lower than that reported in controlled clinical trials. Orthokeratology also allows for good daytime visual function, although it requires strict hygiene due to the risk of infectious complications and the need for regular follow-up visits. Furthermore, the long-term safety of the cornea after several years remains insufficiently studied, and discontinuation of treatment may be associated with reflex axial lengthening. Low-dose atropine remains the gold standard for the pharmacological treatment of myopia in children. Clinical trials such as ATOM2 and LAMP have confirmed that low concentrations, particularly 0.05%, provide a good balance between efficacy and tolerability while minimizing rebound effects after treatment ends. Combination therapy using atropine and optical methods can further improve treatment outcomes. However, recurrence of myopia after treatment discontinuation remains a significant clinical concern, especially at higher concentrations, and long-term treatment strategies are still under investigation. New therapies, including repeated low-intensity red light therapy, violet light exposure, bright light therapy, and artificial intelligence-based, are promising technologies for myopia treatment. However, further long-term studies are necessary to confirm their safety and clinical efficiency.

Current international consensus statements, including those published by the IMI, emphasize a stepwise and evidence-based approach to myopia management. Behavioral interventions, such as increased outdoor exposure, are recommended for all children, while optical and pharmacological interventions are primarily reserved for progressive cases. A synthesis of evidence from Cochrane reviews suggests the highest certainty of benefit for optical interventions based on atropine and defocus, while lifestyle modifications provide moderate preventive effects. However, variability in study design, ethnicity, and outcome measures limits direct comparison of these interventions.

Furthermore, several real-world limitations impact the effectiveness of current myopia control strategies. Socioeconomic factors, the cost of optical devices, and limited access to specialized eye care can limit the implementation of advanced treatments, especially in low-resource settings. Furthermore, medication adherence, especially in the pediatric population, remains a key factor for long-term success, but is often suboptimal outside of clinical trial settings.

## 6. Conclusions

Taking into consideration the number of available methods for controlling the progression of myopia in pediatric patients along with emerging new approaches and clinical trials, selecting the single most effective strategy remains challenging. Among currently available interventions, the strongest level of evidence supports low-dose atropine (particularly 0.05%) and optical defocus-based strategies such as DIMS/HALT spectacle lenses and orthokeratology, which consistently demonstrate clinically significant reductions in axial elongation across randomized controlled trials and meta-analyses. Nevertheless, it is paramount to introduce those therapies as early as possible so that we obtain more beneficial treatment. Additionally, the choice of an appropriate method should be individualized and based on the current condition of the visual system along with patients’ special needs and their lifestyle. Interventions such as repeated low-level red light therapy, violet light exposure, bright light therapy, and AI-based diagnostic or predictive models remain experimental or investigational, as current evidence is limited by short follow-up periods or lack of long-term safety data. Also, the goal of recent trials is to not only increase the effectiveness of treatment but also minimize potential side effects. Due to the massive escalation of myopia, it is paramount to explore and extend new treatments and strive to improve the quality of life. A wide range of current methods for slowing the progression of myopia highlights a dynamic development in this medical field and the increasingly promising prospects for future treatment strategies. Future research should focus on large-scale, multicenter randomized controlled trials with long-term follow-up, head-to-head comparisons of available interventions, and development of standardized clinical guidelines enabling more precise, evidence-based treatment selection in pediatric myopia. From a clinical perspective, the early detection of progressive myopia, regular monitoring of the axial length, and implementation of evidence-based interventions remain the key practical recommendations for clinicians managing pediatric patients.

## Figures and Tables

**Table 1:** Comparison of currently available myopia control interventions.

Intervention	Efficacy	Recommended Age	Safety/Limitations	Patient Adherence	Relative Cost
Increased outdoor activity	Low–moderate (mainly preventive)	All children	Limited efficacy in established myopia	High	Low
Defocus spectacle lenses (DIMS, HALT, CARE)	High (≈50–67%)	6–18 years	Requires full-time wear	High	Moderate
Soft multifocal contact lenses	Moderate–high (≈30–60%)	8–18 years	Lens care required, risk of infection	Moderate	Moderate–high
Orthokeratology	High (≈36–52% reduction in AL elongation)	6–16 years	Overnight lens wear, microbial keratitis risk	Moderate	High
Orthokeratology + low-dose atropine	Very high (>50%, superior to monotherapy)	6–16 years	More complex treatment	Moderate	Very high

## Data Availability

No new data were created or analyzed in this study. Data sharing is not applicable to this article.

## Abbreviations

The following abbreviations are used in this manuscript:

AL	Axial Length
ATOM2	Atropine for the Treatment of Childhood Myopia 2
BLT	Bright Light Therapy
CARE	Cylindrical Annular Refractive Elements
CFP	Color Fundus Photography
CHAMP	Childhood Atropine for Myopia Progression
D	Diopter
DIMS	Defocus Incorporated Multiple Segments
EDOF	Extended Depth Of Focus
FDM	Form Deprivation Myopia
HALT	Highly Aspherical Lenslet Target
IMI	International Myopia Institute
LAMP	Low-Concentration Atropine for Myopia Progression
LORIC	The Longitudinal Orthokeratology Research in Children
LRLR	Low-Level Red Light
OK	Orthokeratology
RGP	Rigid Gas-Permeable (lenses)
RPH	Relative Peripheral Hyperopia
SD-OCT	Spectral-Domain Optical Coherence Tomography
SE	Spherical Equivalent
SER	Spherical Equivalent Refraction
SFCT	Subfoveal Choroidal Thickness
SNP	Single-Nucleotide Polymorphism
TGF-β	Transforming Growth Factor-Beta
TO-SEE	Toric Orthokeratology for Slowing Eyeball Elongation
WHO	World Health Organization
